# Leptin and adiponectin DNA methylation levels in adipose tissues and blood cells are associated with BMI, waist girth and LDL-cholesterol levels in severely obese men and women

**DOI:** 10.1186/s12881-015-0174-1

**Published:** 2015-05-01

**Authors:** Andrée-Anne Houde, Cécilia Légaré, Simon Biron, Odette Lescelleur, Laurent Biertho, Simon Marceau, André Tchernof, Marie-Claude Vohl, Marie-France Hivert, Luigi Bouchard

**Affiliations:** Department of Biochemistry, Université de Sherbrooke, Sherbrooke, QC Canada; ECOGENE-21 and Clinical Research Center and Lipid Clinic, Chicoutimi Hospital, Saguenay, QC Canada; Quebec Heart and Lung Institute, Quebec, Canada; Department of Surgery, Laval University, Quebec, Canada; Axe Endocrinologie et Néphrologie, Centre de recherche du CHU de Québec, Quebec, QC Canada; Department of Food Science and Nutrition, Laval University, Québec, QC Canada; Institute of Nutrition & Functional Foods, Université Laval, Quebec, QC Canada; Department of Medicine, Division of Endocrinology, Université de Sherbrooke, Sherbrooke, QC Canada; Department of Population Medicine, Harvard Pilgrim Health Care Institute, Boston, MA USA; General Medicine Division, Massachusetts General Hospital, Boston, MA USA

**Keywords:** Subcutaneous adipose tissue, Visceral adipose tissue, Epigenetic, Metabolic complications

## Abstract

**Background:**

Leptin (*LEP*) and adiponectin (*ADIPOQ*) genes encode adipokines that are mainly secreted by adipose tissues, involved in energy balance and suspected to play a role in the pathways linking adiposity to impaired glucose and insulin homeostasis. We have thus hypothesized that *LEP* and *ADIPOQ* DNA methylation changes might be involved in obesity development and its related complications. The objective of this study wa**s** to assess whether *LEP* and *ADIPOQ* DNA methylation levels measured in subcutaneous (SAT) and visceral adipose tissues (VAT) are associated with anthropometric measures and metabolic profile in severely obese men and women. These analyses were repeated with DNA methylation profiles from blood cells obtained from the same individuals to determine whether they showed similarities.

**Methods:**

Paired SAT, VAT and blood samples were obtained from 73 severely obese patients undergoing a bioliopancreatic diversion with duodenal switch. *LEP* and *ADIPOQ* DNA methylation and mRNA levels were quantified using bisulfite-pyrosequencing and qRT-PCR respectively. Pearson’s correlation coefficients were computed to determine the associations between *LEP* and *ADIPOQ* DNA methylation levels, anthropometric measures and metabolic profile.

**Results:**

DNA methylation levels at the *ADIPOQ* gene locus in SAT was positively associated with BMI and waist girth whereas *LEP* DNA methylation levels in blood cells were negatively associated with body mass index (BMI). Fasting LDL-C levels were found to be positively correlated with DNA methylation levels at *LEP*-CpG11 and -CpG17 in blood and SAT and with *ADIPOQ* DNA methylation levels in SAT (CpGE1 and CpGE3) and VAT (CpGE1).

**Conclusions:**

These results confirm that *LEP* and *ADIPOQ* epigenetic profiles are associated with obesity. We also report associations between LDL-C levels and both *LEP* and *ADIPOQ* DNA methylation levels suggesting that LDL-C might regulate their epigenetic profiles in adipose tissues. Furthermore, similar correlations were observed between LDL-C and *LEP* blood DNA methylation levels suggesting a common regulatory pathway of DNA methylation in both adipose tissues and blood.

**Electronic supplementary material:**

The online version of this article (doi:10.1186/s12881-015-0174-1) contains supplementary material, which is available to authorized users.

## Background

The incidence of obesity and its related disorders (dyslipidemia, hypertension, type 2 diabetes and cardiovascular diseases) has been constantly increasing in the last few decades leading to a global obesity epidemic [1,2]. Although the heritability estimates for obesity range between 6%-85% depending on the trait assessed [3], the obesity-related genetic variants identified have so far explained less than 2% of the heritability of obesity [4,5]. Hence, it is unlikely that the current obesity epidemic is solely caused by genetic variations. The interactions between the gene variants and the components of our obesogenic environment are very likely contributing to the increasing obesity rates.

Genes are known to adapt to the environment through epigenetic modifications [6,7] among other mechanisms. Epigenetics refers to the molecular mechanisms regulating gene expression without affecting the DNA sequence [8]. DNA methylation, the most understood epigenetic mark, primarily occurs on the cytosine upstream of a guanine (dinucleotide CpG) and is catalyzed by the DNA methyltransferases (DNMTs) [9]. The methylation of cytosines in a CpG context has been shown to be sensitive to environmental stimuli including *in utero* [10-12] and post-natal environmental conditions [13,14].

Epigenetic modifications contribute to the pathogenesis of obesity and its obesity-related metabolic complications. Indeed, studies have reported that DNA methylation levels at candidate gene loci related to obesity and metabolic diseases are impaired in blood and adipose tissues of obese patients [15,16] and in low weight loss responder to diet and exercise interventions [17-20]. The leptin (*LEP*) and adiponectin (*ADIPOQ*) genes are probably those that have warranted the most attention so far. These genes encode for leptin and adiponectin proteins, which are mainly synthesized and secreted by the adipocytes. Leptin plasma concentrations are increased in obese subjects (leptin resistance is suspected) and has both anorexigenic and proinflammatory properties [21,22], whereas adiponectin improves insulin sensitivity, exerts anti-inflammatory actions and its secretion is significantly reduced in obesity [21,22].

We have previously reported that maternal hyperglycaemia (2 h post-oral glucose tolerance test (OGTT)) during the second trimester of pregnancy is associated with decreased placental DNA methylation levels at *LEP* [23] and *ADIPOQ* [24] gene loci suggesting that epigenetic adaptations could be involved in fetal metabolic programming and increase newborn lifelong susceptibility to obesity and metabolic disorders. Other groups have reported that whole blood *LEP* DNA methylation levels are negatively associated with birth weight and child BMI at 17 months [25]. In addition, DNA methylation levels at the *LEP* and *ADIPOQ* gene promoters in blood were recently found to be lower in obese and insulin resistant adolescents [26]. Altogether these results suggest that *LEP* and *ADIPOQ* DNA methylation profiles might be involved in the pathology of obesity and cardiometabolic diseases. Nevertheless, *LEP* and *ADIPOQ* epigenetic profiles in adipose tissue and their associations with obesity and obesity-associated metabolic perturbations have not been assessed so far. Accordingly, we hypothesized that decreased *LEP* DNA methylation and increased *ADIPOQ* DNA methylation in adipose tissue could lead to higher degree of obesity and pro-inflammatory state, dyslipidemia, hypertension and insulin resistance. Henceforth, the objective of this study was to determine whether *LEP* and *ADIPOQ* DNA methylation levels in subcutaneous (SAT) and visceral (VAT) adipose tissues were associated with obesity and obesity-related complications severely obese men and women. SAT and VAT were both analysed because they show specific gene expression profiles (ex. *LEP* and *ADIPOQ*) [27,28] and associations with cardiovascular risk factors [29]. Moreover, we tested whether adipokine epigenetic profiles in blood reflect those in adipose tissues and whether they could be used as proxies.

## Methods

### Subjects

Blood, SAT and VAT samples were obtained from 33 men and 40 premenopausal women (BMI >40 kg/m^2^) undergoing bioliopancreatic diversion with duodenal switch to treat obesity. They were selected based on the fact that they were free of treatment for dyslipidemia, hypertension and diabetes. The surgical and sampling procedures have been described previously [30,31]. All participants provided a written informed consent before their inclusion in the study, and all clinical data were denominalized. This project was performed in collaboration with the Tissue bank for the study of obesity and its complications at the Institut Universitaire de Cardiologie et de Pneumologie de Québec. The project was approved by this institution’s and the Université Laval’s ethics committees and was conducted in accordance with the Declaration of Helsinki.

### Nucleic acid extraction

DNA was purified from whole blood samples with the Gentra Puregene Blood Kit (Qiagen, Valencia, CA). DNA and RNA from SAT and VAT were extracted as previously described [32]. RNA quality was assessed with Agilent 2100 Bioanalyzer RNA Nano Chips (Agilent Technologies, Santa Clara, CA). Three RNA samples from SAT and VAT had low RNA integrity numbers (RIN < 6.0) and were excluded from the analysis. The other RNA samples in SAT and VAT showed a high quality with mean RIN values of 8.0 ± 0.8 and 8.3 ± 0.6 respectively. RNA samples were not available for blood samples.

### DNA methylation analyses and genotyping

DNA methylation levels at CpG sites were assessed using pyrosequencing (Pyromark Q24, QIAGEN-Biotage). Combined with the NaBis DNA treatment, pyrosequencing is a quantitative real-time sequencing technology that allows to measure DNA methylation levels (%) at a single cytosine (CpG) of a given genomic region. The NaBis treatment of DNA (EpiTect Bisulfite Kit, Qiagen) specifically converts unmethylated cytosines into uracil, while the methylated cytosines are protected from this transition, creating a cytosine/thymine polymorphism. Once treated, NaBis-DNA is amplified (Pyromark PCR kit, Qiagen), and the cytosine and thymine alleles are quantified by pyrosequencing [33]. Specific PCR and pyrosequencing primer pairs for *LEP* and *ADIPOQ* DNA methylation analyses are described in Additional files 1 and 2.

Genotyping of the *LEP* single nucleotide polymorphism (SNP) rs2167670 was performed in the three tissues using pyrosequencing as reported before [34]. The rs2167270 genotype was identical in all three tissues analysed for the 73 patients. Genotype frequencies (GG: 30 (41.1%); GA: 35 (47.9%) and AA: 8 (11.0%)) were found to be under Hardy-Weinberg equilibrium (*p* > 0.05). Carriers of the minor A allele (GA/AA) were grouped together for statistical analysis purposes as the number of homozygous AA was very low.

### *LEP* and *ADIPOQ* mRNA measurements

mRNA levels in SAT and VAT were quantified by quantitative real-time PCR (qRT-PCR). Complementary DNA (cDNA) was generated from total RNA using a random primer hexamer provided with the High Capacity cDNA Archive Kit from Applied Biosystems (Foster City, CA). Equal amounts of cDNA were run in duplicate and amplified in a 20 μL reaction containing 10 μL of Universal PCR Master Mix (Applied Biosystems). Primers and Taqman probes were obtained from Applied Biosystems (*LEP*: Hs00174877_m1 and *ADIPOQ*: Hs00605917_m1). The *Glyceraldehyde 3-phosphate dehydrogenase* (*GAPDH:* Hs99999905_m1; Applied Biosystems) housekeeping gene was amplified in parallel. *LEP, ADIPOQ* and *GAPDH* amplifications were performed using the Applied Biosystems 7500 Real Time PCR System, as recommended by the manufacturer (Applied Biosystems). *LEP* and *ADIPOQ* mRNA (Ct) levels were quantified relative to change in *GAPDH* gene expression (Ct). *GAPDH*/*ADIPOQ* and *GAPDH/LEP* Ct ratios (1/x) were used for the analysis.

### Statistical analyses

The mean DNA methylation of the 21 CpGs analysed in the proximal promoter CpG island of *LEP* gene (Additional file 3) was first computed (*LEP*-Mean) and analysed. Moreover, since we [34] and other groups [35-37] have previously reported that CpGs located between CpG7 to CpG17 are of first interest for *LEP* gene expression regulation through DNA methylation changes, we analysed these CpGs individually to identify those more likely to be regulatory. Out of these 8 CpGs, we excluded the CpG sites that were found to be unmethylated (≤10.0%) or hypermethylated (≥90%) as they show low DNA methylation variability and are thus unlikely to explain the phenotypic variability. Hence, CpG12 to CpG16 were not analysed further in adipose tissues and blood (Additional file 3).

At *ADIPOQ* gene locus*,* CpG island A and C were either unmethylated (≤10.0%) or hypermethylated (≥90%) and were thus not further analysed (Additional file 4). Mean DNA methylation levels were computed for CpG island E in SAT, VAT (*ADIPOQ*-Mean). CpGE1 and CpGE3 were analysed individually in SAT and VAT, whereas CpGE2 (hypermethylated) was not analysed further. In blood, CpGE3 was the only one analysed as the *ADIPOQ*-Mean, and CpGE1 and CpGE2 were found to be hypermethylated (Additional file 4).

The normal distribution of all variables was assessed using a Kolmogorov-Smirnov test. Fasting triglyceride (TG), C-reactive protein (CRP) and both fasting glucose and DNA methylation levels at *ADIPOQ*-CpGE3 locus in blood were found to be normally distributed after they were log10-transformed and ranked respectively. The associations between adipokine genes DNA methylation levels, anthropometric measures and metabolic profile were assessed with partial Pearson’s correlations. The partial Pearson correlations were also used to determine the relationship between fasting low-density lipoprotein cholesterol (LDL-C) levels, *LEP* DNA methylation and mRNA levels in SAT. Pearson’s correlation coefficients were adjusted for the following covariates when appropriate: sex, age, and waist circumference. Waist circumference is a stronger cardiometabolic disease risk marker and was preferred to BMI as a covariate [38,39]. Moreover, all results remained unchanged after consideration of smoking status. Two-sided *p-*values ≤ 0.05 were considered statistically significant. The statistical analyses were performed with the IBM SPSS Statistics 20 software (release 20.0.0, SPSS, Chicago, Il, USA).

## Results

The characteristics of the subjects included in this study are shown in Table 1. All subjects were severely obese (obese class III [40]) with BMI ranging from 40.0 to 60.0 kg/m^2^. The patients were slightly hypertensive but had generally good metabolic health without diabetes or dyslipidemia [41,42].

**Table 1:** **Characteristics of the subjects studied (n = 73)**

	**Mean ± SD**	**Range (min-max)**
Men (%)	33 (45.2%)	-
Age (years)	34.7 ± 7.1	21.4 – 53.8
BMI (kg/m^2^)	49.6 ± 6.0	40.0 – 60.0
Hip circumference (cm)	147.7 ± 14.0	123.0 – 193.0
Waist circumference (cm)	139.3 ± 16.6	99.0 – 180.0
Waist hip ratio	0.95 ± 0.11	0.68 – 1.17
Fasting glucose (mmol/l)^a^	5.51 ± 1.05	4.00 – 8.70
Systolic blood pressure (mm Hg)	137.0 ± 14.0	101.0 – 183.0
Diastolic blood pressure (mm Hg)	86.0 ± 10.0	57.0 – 108.0
TC (mmol/l)	4.87 ± 0.85	3.22 – 6.92
LDL-C (mmol/l)	2.96 ± 0.76	1.20 – 5.40
HDL-C (mmol/l)	1.17 ± 0.27	0.70 – 2.12
TC/HDL-C	4.32 ± 1.24	0.98 – 7.56
TG^a^ (mmol/l)	1.52 ± 0.98	0.52 – 6.89
CRP^a^ (mg/L) (n = 53)	8.2 ± 10.2	2.0 – 54.9
Current smokers	17 (23.3%)	-

BMI, body Mass Index; TC, total cholesterol; LDL-C, low-density lipoprotein-cholesterol; HDL-C, high-density lipoprotein-cholesterol; TC/HDL-C, total cholesterol to high-density lipoprotein-cholesterol ratio; TG, triglycerides; CRP, c-reactive protein.

^*a*^Geometric mean*.*

### *LEP* and *ADIPOQ* DNA methylation and anthropometric variables

**Figure 1:**
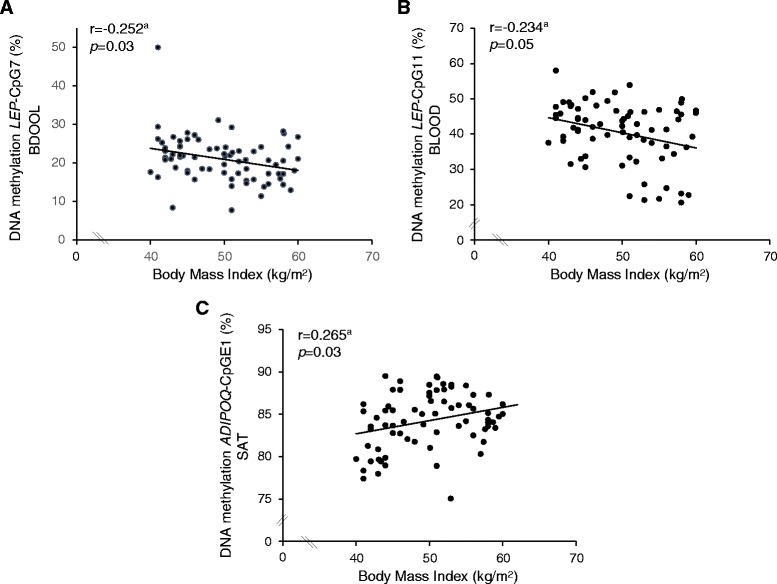
*LEP* and *ADIPOQ* DNA methylation levels according to body mass index (BMI) in severely obese patients. *LEP* DNA methylation levels at CpG7 **(A)** and CpG11 **(B)** in blood were associated with BMI. In SAT, *ADIPOQ* DNA methylation levels at CpGE1 were positively associated with BMI **(C)**. ^a^Adjusted for age and sex (n = 73).

To determine whether *LEP* and *ADIPOQ* epigenetic profiles are involved in the pathogenesis of obesity and cardiometabolic complications, we first assessed the associations between *LEP* and *ADIPOQ* DNA methylation levels in adipose tissues and blood and obesity-related anthropometric measures. The mean DNA methylation levels of *LEP* promoter CpG island were found to be negatively correlated with BMI (r = −0.328; *p* = 0.005) and hip circumference (r = −0.230; *p* = 0.05) (Pearson’s correlation coefficients adjusted for age and sex) (Additional file 5). To identify the CpGs that are more likely to be regulatory, *LEP*-CpG7, *LEP*-CpG11 and *LEP*-CpG17 were analysed independently. Negative correlations were observed between BMI and *LEP*-CpG7 (r = −0.252; *p* = 0.03) and *LEP*-CpG11 (r = −0.234; *p* = 0.05) (Figure 1-Panel A and B). As obesity-related proinflammatory response is associated with higher plasma leptin levels, we also assessed whether *LEP* DNA methylation levels in blood cells are associated with circulating levels of the pro-inflammatory CRP. Lower *LEP*-Mean and *LEP*-CpG7 DNA methylation levels in blood were associated with increased levels of CRP (*LEP*-Mean: r = −0.285; *p* = 0.05 and *LEP*-CpG7: r = −0.397, *p* = 0.004, n = 53) (Additional file 6). Of note, the correlations reported between *LEP* DNA methylation levels in blood and BMI were no longer significant after adjusting for CRP levels (*LEP*-Mean: r = −0.237; *p* = 0.10; *LEP*-CpG7: r = −0.151; *p* = 0.294 and *LEP*-CpG11: r = −0.155; *p* = 0.283 (n = 53)).

**Figure 2:**
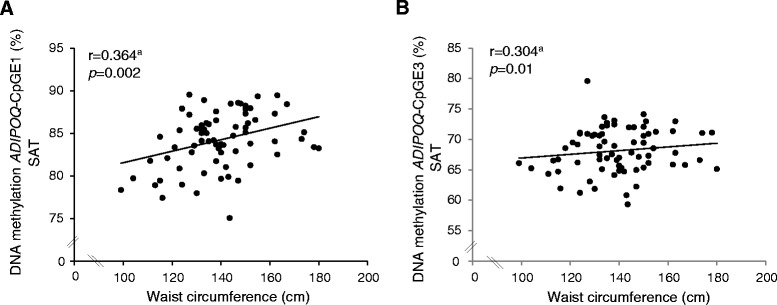
*ADIPOQ* DNA methylation levels according to waist circumference in severely obese patients. *ADIPOQ* DNA methylation levels at CpGE1 **(A)** and CpGE3 **(B)** in SAT were positively correlated with waist circumference. ^a^Adjusted for age and sex (n = 73).

*ADIPOQ* DNA methylation levels in SAT were found correlated with BMI (*ADIPOQ*-Mean: r = 0.250; *p* = 0.04 and *ADIPOQ*-CpGE1: r = 0.265; *p* = 0.03) (Figure 1-Panel C) (Additional file 5) and waist circumference (*ADIPOQ*-Mean: r = 0.361; *p* = 0.002; *ADIPOQ*-CpGE1: r = 0.364; *p* = 0.002 and *ADIPOQ*-CpGE3: r = 0.304; *p* = 0.01) (Figure 2-Panel A and B) (Additional file 5). Moreover, DNA methylation levels at *ADIPOQ-*CpGE3 locus in SAT were positively correlated with hip circumference (r = 0.234; *p* = 0.05) (Additional file 5). No additional association was observed between *LEP* and *ADIPOQ* DNA methylation levels and anthropometric variables (Additional file 5).

### *LEP* and *ADIPOQ* DNA methylation and obesity-related complications

We next determined whether *LEP* and *ADIPOQ* DNA methylation levels in adipose tissue and blood were associated with obesity-related complications including dyslipidemia, hyperglycemia and hypertension. Fasting low-density lipoprotein-cholesterol (LDL-C) levels were positively correlated with *LEP*-Mean DNA methylation levels in VAT (r = 0.273; *p* = 0.02) and *ADIPOQ*-Mean DNA methylation levels in SAT (r = 0.268; *p* = 0.03) and VAT (r = 0.245; *p* = 0.04) (Table 2). Analyses of individual CpG sites revealed positive correlations between LDL-C levels and DNA methylation levels at *LEP*-CpG17 locus in blood (r = 0.234; *p* = 0.05) and SAT (r = 0.391; *p* = 0.001) (Table 2). At the *ADIPOQ* gene loci, CpGE1 (r = 0.236; *p* = 0.05) and CpGE3 (r = 0.292; *p* = 0.01) in SAT and CpGE1 in VAT (CpGE1: r = 0.267; *p* = 0.03) were also positively correlated with fasting LDL-C levels (Table 2). A trend for association was also observed between LDL-C and DNA methylation levels at *LEP*-CpG11 in blood and SAT (Table 2). DNA methylation levels at the *ADIPOQ* gene loci (*ADIPOQ*-Mean and *ADIPOQ*-CpGE3) in SAT and at *LEP*-CpG7 in VAT were associated with fasting total cholesterol (TC) and high-density lipoprotein-cholesterol (HDL-C), respectively (Additional file 6). Adipokine genes DNA methylation levels were not found to be associated with fasting glucose levels and either systolic or diastolic blood pressure (Additional file 6).

**Table 2:** **Pearson correlation coefficients between fasting LDL-C levels and both** ***LEP*** **and** ***ADIPOQ*** **DNA methylation and mRNA levels in subcutaneous (SAT) and visceral adipose tissues (VAT)**

	**LDL-C levels (mmol/L) Adjusted for age and sex**		**LDL-C levels (mmol/L) Adjusted for age, sex and WG**	
	**r**	***P***	**r**	***p***
**BLOOD**				
*LEP*-CpG7	0.133	0.27	0.122	0.31
*LEP*-CpG11	0.222	0.06	0.206	0.09
*LEP*-CpG17	**0.239**	**0.04**	0.234	0.05
*LEP-*Mean	0.178	0.138	0.162	0.18
*ADIPOQ-CpGE3* ^*a*^	0.161	0.17	0.149	0.22
**SAT**				
*LEP*-CpG7	0.090	0.46	0.108	0.37
*LEP*-CpG11	0.215	0.07	0.231	0.06
*LEP*-CpG17	**0.380**	**0.001**	**0.391**	**0.001**
*LEP-*Mean	0.190	0.11	0.218	0.07
*LEP* mRNA levels	−0.113	0.35	−0.098	0.42
*ADIPOQ-CpGE1*	0.176	0.14	**0.236**	**0.05**
*ADIPOQ-CpGE3*	**0.242**	**0.04**	**0.292**	**0.01**
*ADIPOQ-*Mean	0.206	0.08	**0.268**	**0.03**
*ADIPOQ* mRNA levels	0.024	0.85	0.009	0.94
**VAT**				
*LEP*-CpG7	0.130	0.28	0.131	0.28
*LEP*-CpG11	0.158	0.19	0.169	0.16
*LEP*-CpG17	0.114	0.34	0.127	0.30
*LEP-*Mean	0.256	0.03	0.273	0.02
*LEP* mRNA levels	0.160	0.18	0.034	0.78
*ADIPOQ-CpGE1*	**0.270**	**0.02**	**0.267**	**0.03**
*ADIPOQ-CpGE3*	0.188	0.12	0.181	0.14
*ADIPOQ-*Mean	**0.250**	**0.04**	**0.245**	**0.04**
*ADIPOQ* mRNA levels	−0.111	0.37	−0.121	0.33

WG,waist girth.

^a^Results obtained after rank transformation of DNA methylation levels.

Values in bold type are statistically significant: *p* ≤ 0.05.

### *LEP* and *ADIPOQ* DNA methylation and mRNA levels in SAT and VAT, and obesity-related complications

To further explore the functionality of the associations reported in the two previous sections, we verified whether *LEP* and *ADIPOQ* gene expression and DNA methylation levels in SAT and VAT were correlated with phenotypic variability.

We have previously reported that *LEP*-CpG17 DNA methylation levels are negatively associated with *LEP* mRNA levels in SAT, specifically in carriers of the rs2167270 A alleles (GA/AA genotypes; r = −0.367, *p* = 0.02) [34] (Table 3). The *LEP* genotypes were thus taken into account. LDL-C levels were found to be negatively correlated with *LEP* mRNA levels (r = −0.317; *p* = 0.04) in rs2167270 A allele carriers only (Table 3). Of note, the correlation between *LEP* mRNA and LDL-C levels was no longer significant after adjusting Pearson’s correlation coefficient for *LEP*-CpG17 DNA methylation levels (r = −0.205; *p* = 0.21) (Table 3).

**Table 3:** **Pearson correlation coefficients between low-density lipoprotein cholesterol (LDL-C) levels,** ***LEP*** **mRNA and** ***LEP*** **gene CpG17 DNA methylation levels in subcutaneous adipose tissue (SAT) according to the rs2167270 genotype**

	***LEP*** **mRNA levels**			***LEP*** **-CpG17 DNA methylation levels**		
	**ALL**	**GG**	**GA/AA**	**ALL**	**GG**	**GA/AA**
	**n = 73**	**n = 30**	**n = 43**	**n = 73**	**n = 30**	**n = 43**
**LDL-C levels**	−0.097	0.142	−0.317^*^	0.370^**^	0.400^*^	0.384^**^
Adjusted for sex and waist girth						
**LDL-C levels**	−0.035	0.138	−0.205	-	-	-
Adjusted for sex, waist girth and *LEP*-CpG17 DNA methylation levels						
**LDL-C levels**	-	-	-	0.360^**^	0.399^*^	0.303^†^
Adjusted for sex, waist girth and *LEP* mRNA levels						
***LEP*** **-CpG17 DNA methylation levels**	−0.177	0.038	−0.367^*^	-	-	-
Adjusted for sex						
***LEP*** **-CpG17 DNA methylation levels**	−0.152	−0.021	−0.282^†^	-	-	-
Adjusted for sex and LDL-C levels						

Pearson correlations are statistically significant: ^†^ *p* ≤ 0.10; ^*^ *p* ≤ 0.05*;* ^**^ *p* ≤ 0.01.

In SAT, DNA methylation levels at *ADIPOQ*-CpGE3 were negatively correlated with *ADIPOQ* mRNA levels (r = 0.257; *p* = 0.03). *ADIPOQ* mRNA levels in SAT or VAT were not associated with BMI, waist girth, hip circumference or LDL-C levels (Table 2 and Additional file 5).

## Discussion

In the current study, we report that *LEP* and *ADIPOQ* DNA methylation levels, measured in paired adipose tissues and blood samples, are associated with obesity-related anthropometric variables and fasting LDL-C levels.

We first demonstrated that lower *LEP* promoter DNA methylation levels in blood cells are associated with higher BMI. Several cross-sectional studies have reported lower *LEP* DNA methylation levels in blood cells of newborns and children with higher birth weight and BMI [25], in obese and insulin resistant adolescents [26] and obese women [43]. Our findings are also concordant with fetal metabolic programming studies in humans and rodent models which have lately shown that lower *LEP* DNA methylation levels in blood, placenta, VAT, liver and muscle are associated with a risk for obesity and metabolic diseases in the offspring [10,44,23]. It is thus tempting to speculate on whether the *LEP* epigenetic profile we are reporting in blood was established *in utero* and might have been involved in the development of obesity in the patients of the current study. However, we would have expected that the *LEP* epigenetic signature that we are describing in blood would also have been present in adipose tissues, if programmed *in utero*. Because the correlations between *LEP* DNA methylation levels and BMI were attenuated after adjusting for CRP levels, we hypothesized that the obesity-related proinflammatory response *per se* contributed to the alteration of *LEP* epigenetic profile in blood cells. Leptin is mainly produced by adipose tissues but is also expressed by peripheral blood mononuclear cells [45]. It is also recognized that plasma leptin and CRP levels are upregulated in obesity and proinflammatory states and closely related [46]. Plasmatic elevation of CRP levels observed in obese subjects has been associated with higher plasma concentrations and leptin resistance [46]. Hence, increased CRP levels might, to some extent, regulate plasmatic leptin concentrations and leptin resistance via the modification of *LEP* DNA methylation levels. Unfortunately, because blood cell counts were not available, we cannot exclude that *LEP* DNA methylation variability and the associations reported with CRP and BMI might be partially attributed to blood cell composition changes between samples. However, if cellular composition changes impact the results, this effect is likely modest as highlighted by Talens *et al*. [47]. DNA methylation regulation is (partly) tissue-specific and might explain the discrepancy between the associations reported in blood and adipose tissues [48,49]. Also, we cannot exclude that *LEP* epigenetic profile of these “metabolically-healthy” individuals might have been re-programmed by the post-natal environmental and stochastic factors.

We also report that *ADIPOQ* DNA methylation levels in SAT are positively associated with BMI and waist circumference. *ADIPOQ* DNA methylation levels at CpGE3 are also associated with *ADIPOQ* mRNA levels in SAT. This suggests that epigenetic modifications at *ADIPOQ* gene locus are functional and could potentially be involved in the pathogenesis of impaired glucose tolerance and insulin resistance associated with obesity. Nevertheless, we cannot exclude that higher *ADIPOQ* DNA methylation levels would have led to higher degree of obesity. In accordance with this hypothesis, we have lately reported that placental *ADIPOQ* DNA methylation levels are impaired following exposure to maternal glucose 2 h post-OGTT at second trimester of pregnancy suggesting that *ADIPOQ* epigenetic profile can increase susceptibility to obesity in the newborn [24]. Whether *ADIPOQ* epigenetic profile in SAT is established before or after the onset of obesity will need to be confirmed, but is clearly of strong interest.

Interestingly, *LEP* DNA methylation levels in blood, SAT and VAT, and *ADIPOQ* DNA methylation levels in SAT and VAT were both found to be positively associated with LDL-C levels suggesting a common epigenetic regulation independently of their biological roles although these two adipokines are oppositely regulated. In addition, we report that the association between LDL-C and *LEP* mRNA levels are partially dependent on *LEP* rs2167270 genotype and DNA methylation levels at CpG17. I*n vitro* studies have shown that the incubation of endothelial cells with LDL or oxidized-LDL increases DNA methylation levels at specific gene loci through the induction of DNA methyltransferase 1 (DNMT1) expression and activity [50,51]. Interestingly, the use of statins, the most prescribed lipid lowering drugs, is associated with the down regulation of DNMT activity and demethylation of *BMP2* promoter in mice and cell culture models [52]. Whether the effect of statins is direct (DNMT activity) or mediated through LDL-C lowering needs to be further investigated. Still, our results and those from these previous studies suggest that LDL-C might be involved in the regulation of *LEP* and *ADIPOQ* gene DNA methylation levels with possible impacts on mRNA transcription, and that, independently of their biological roles. It is recognized that the treatment of dyslipidemia with statins increases insulin resistance and the risk for type 2 diabetes [53,54]. The modifications of LDL-C levels and adipokines epigenetic profile by statins might be one of the mechanisms contributing to the insulin resistance state. Consequently, it will be of great clinical interest to confirm the directionality of these associations and to further investigate their respective mechanisms of regulation through epigenetic changes.

The identification of DNA methylation surrogate measures in readily accessible tissue remains an important challenge in epigenetic epidemiology [55,11]. In the current study, paired adipose tissues and blood samples were analysed and tested to assess whether they show DNA methylation profile similarities at adipokine gene loci. DNA methylation levels at *LEP*-CpG11 and CpG17 were found to be associated with LDL-C levels in both SAT and blood samples. These two CpGs were recently identified by our group among the most promising CpG sites to be used in blood as a surrogate for *LEP* DNA methylation in adipose tissues [34]. DNA methylation levels at CpG11 and CpG17 were found to be modestly but significantly correlated between SAT and blood (r = 0.43, p < 0.01; r = 0.58, p < 0.01 respectively) [34]. These two CpGs are respectively within *C/EBP* and *SP1* transcription factor binding sites (TFBS), both involved in *LEP* gene expression regulation [56,35]. The correlations reported between *LEP*-CpG11 and *-*CpG17 DNA methylation and LDL-C levels in SAT and blood suggesting that DNA methylation regulation by LDL-C at these two CpG sites is common to both tissues. Moreover, as these associations are similar in SAT and blood, they are very likely independent of cellular count. The current results thus support that *LEP*-CpG11 and -CpG17 DNA methylation levels in blood are potential relevant surrogates for SAT DNA methylation levels.

The strengths of the current study include analyses of two adipokines central in energy metabolism regulation in two adipose tissue compartments and in blood. Blood is the most clinically accessible tissue whereas adipose tissues are those biologically relevant for the study of obesity and adipokine epigenetic regulation. Access to adipose tissues remains restricted, and this study provides insights on the use of blood as a potential surrogate tissue in epigenetic epidemiology. None of our participants were taking medication to treat any of the metabolic syndrome components and were on average metabolically fit. This metabolically-healthy obese population has a unique obesity phenotype and metabolism, and *LEP* and *ADIPOQ* DNA methylation profiles might be distinct in these participants. Consequently, the results reported in the current study need to be validated in normal weight populations as well as obese populations with cardiometabolic complications. Moreover, as we are reporting cross-sectional observations, longitudinal and functional studies are needed to determine the causality of the relationships between adipokine DNA methylation, anthropometric variables and plasma LDL-C levels.

## Conclusions

This study provides further evidence that *LEP* DNA methylation levels in blood cells and *ADIPOQ* DNA methylation levels in SAT are associated with obesity-related anthropometric measures. It also suggests that LDL-C, known to regulate DNA methylation processes, could be involved in adipokines’ gene expression regulation through epigenetic changes. Interestingly, similar correlations were observed between *LEP* DNA methylation and LDL-C levels in blood and SAT. This might be of clinical importance considering that the access to biologically active tissues, such as adipose tissues is limited.

## Additional files

Additional file 1:
**PCR and pyrosequencing primers for** ***LEP*** **gene proximal promoter CpG island amplification and pyrosequencing.**
Additional file 2:
**PCR and pyrosequencing primers for** ***ADIPOQ*** **gene CpG islands amplification and pyrosequencing.**
Additional file 3:
**CpG island within** ***LEP*** **gene proximal promoter region.** The CpG sites are numbered according to Bouchard *et al*. 2010 [23]. The underlined CpG sites were epigenotyped in the current study. Associations between *LEP* DNA methylation levels, anthropometric variables and obesity-related complications were assessed at CpG sites for which DNA methylation values are in bold type. The sequence (−11131 to −10563) was numbered relative to the first leptin AUG codon (UCSC Genome Browser (Human Feb. 2009)). Additional file 4:
**CpG islands analysed within** ***ADIPOQ*** **gene locus.** CpG islands A and C were found to be hypomethylated and hypermethylated respectively and were not analysed. Associations between *ADIPOQ* DNA methylation levels, anthropometric variables and obesity-related complications were assessed at CpG sites for which DNA methylation values are in bold type. Additional file 5:
**Pearson correlation coefficients between** ***LEP*** **and** ***ADIPOQ*** **DNA methylation and mRNA levels in blood, subcutaneous (SAT) and visceral adipose tissue (VAT) and anthropometric variables (adjusted for age and sex) (n = 73).**
Additional file 6:
**Pearson correlation coefficients between** ***LEP*** **and** ***ADIPOQ*** **DNA methylation and mRNA levels in blood, subcutaneous (SAT) and visceral adipose tissues (VAT) and cardiometabolic risk factors (adjusted for age, sex and waist circumference) (n = 73).**

## Abbreviations

ADIPOQ: Adiponectin
BMI: Body mass index
CRP: C-reactive protein
DNMT: DNA methyltransferase
HDL-C: High-density lipoprotein cholesterol
LDL-C: Low-density lipoprotein cholesterol
LEP: Leptin
SAT: Subcutaneous adipose tissue
SNP: Single nucleotide polymorphism
TC: Total cholesterol
VAT: Visceral adipose tissue
