# Supplementary material for: Leptin and adiponectin DNA methylation levels in adipose tissues and blood cells are associated with BMI, waist girth and LDL-cholesterol levels in severely obese men and women
Source: BMC Med Genet. 2015 May 1;16:29. doi: 10.1186/s12881-015-0174-1 (PMC4631085; doi:10.1186/s12881-015-0174-1)
Supplement: Additional file 1: — PCR and pyrosequencing primers for LEP gene proximal promoter CpG island amplification and pyrosequencing. [file 12881_2015_174_MOESM1_ESM.pdf]

Additional File 1. Pearson correlation coefficients between *LEP* and *ADIPOQ* DNA methylation and mRNA levels in blood, subcutaneous (SAT) and visceral adipose tissue (VAT) and anthropometric variables (adjusted for age and sex) (n=73).

	**Body Mass Index (kg/m^2)**		**Waist Circumference (cm)**		**Hip Circumference (cm)**		**Waist Hip Ratio**	
	**r**	***p***	**r**	***p***	**r**	***p***	**r**	***p***
**BLOOD**								
LEP-CpG7	**-0.252**	**0.03**	-0.107	0.38	-0.197	0.10	0.116	0.34
LEP-CpG11	**-0.234**	**0.05**	-0.178	0.14	-0.214	0.07	0.060	0.62
LEP-CpG17	-0.151	0.21	-0.065	0.59	-0.157	0.19	0.104	0.39
LEP-Mean	**-0.328**	**0.005**	-0.167	0.16	**-0.230**	**0.05**	0.074	0.54
ADIPOQ-CpGE3[a]	0.131	0.28	-0.152	0.21	-0.026	0.83	-0.104	0.39
**SAT**								
LEP-CpG7	0.059	0.62	0.141	0.24	0.068	0.57	0.082	0.50
LEP-CpG11	0.023	0.85	0.107	0.37	-0.007	0.95	0.137	0.26
LEP-CpG17	-0.091	0.45	0.063	0.60	-0.102	0.40	0.166	0.17
LEP-Mean	0.108	0.37	0.194	0.11	0.140	0.24	0.063	0.60
LEP mRNA levels	0.211	0.08	0.138	0.25	0.228	0.06	-0.090	0.46
ADIPOQ-CpGE1	**0.265**	**0.03**	**0.364**	**0.002**	0.205	0.09	0.168	0.16
ADIPOQ-CpGE3	0.192	0.11	**0.304**	**0.010**	**0.234**	**0.05**	0.054	0.66
ADIPOQ-Mean	**0.250**	**0.04**	**0.361**	**0.002**	0.201	0.09	0.160	0.18
ADIPOQ mRNA levels	-0.074	0.54	-0.128	0.29	-0.022	0.86	-0.101	0.41
**VAT**								
LEP-CpG7	0.107	0.37	0.002	0.98	0.048	0.69	-0.004	0.98
LEP-CpG11	0.045	0.71	0.080	0.505	-0.028	0.82	0.125	0.30
LEP-CpG17	-0.074	0.54	0.099	0.412	-0.007	0.95	0.088	0.46
LEP-Mean	0.037	0.76	0.116	0.33	0.022	0.86	0.101	0.40
LEP mRNA levels	0.119	0.32	0.173	0.15	0.038	0.76	0.142	0.24
ADIPOQ-CpGE1	-0.072	0.55	-0.046	0.71	-0.103	0.39	0.031	0.80
ADIPOQ-CpGE3	-0.141	0.242	-0.076	0.53	-0.058	0.63	-0.047	0.70
ADIPOQ-Mean	-0.105	0.38	-0.057	0.64	-0.106	0.38	0.024	0.84
ADIPOQ mRNA levels	-0.068	0.58	-0.075	0.54	0.003	0.98	-0.101	0.41

[a]Results obtained after rank transformation of the DNA methylation levels
Values in bold type are statistically significant ($p \leq 0.05$)
