# Supplementary material for: Leptin and adiponectin DNA methylation levels in adipose tissues and blood cells are associated with BMI, waist girth and LDL-cholesterol levels in severely obese men and women
Source: BMC Med Genet. 2015 May 1;16:29. doi: 10.1186/s12881-015-0174-1 (PMC4631085; doi:10.1186/s12881-015-0174-1)
Supplement: Additional file 2: — PCR and pyrosequencing primers for ADIPOQ gene CpG islands amplification and pyrosequencing. [file 12881_2015_174_MOESM2_ESM.pdf]

**Additional File 2.** Pearson correlation coefficients between *LEP* and *ADIPOQ* DNA methylation and mRNA levels in blood, subcutaneous (SAT) and visceral adipose tissues (VAT) and cardiometabolic risk factors (adjusted for age, sex and waist circumference) (n=73).

| | **TG[b] (mmo/L)** | | **TC (mmo/L)** | | **HDL-C (mmo/L)** | | **Glucose[a] (mmo/L)** | | **CRP[b] (mg/L ) (n=53)** | | **SBP (mm Hg)** | | **DBP (mm Hg)** | |
|---|---|---|---|---|---|---|---|---|---|---|---|---|---|---|
| | **r** | ***p*** | **r** | ***p*** | **r** | ***p*** | **r** | ***p*** | **r** | ***p*** | **r** | ***p*** | **r** | ***p*** |
| **BLOOD** | | | | | | | | | | | | | | |
| *LEP*-CpG7 | 0.119 | 0.33 | 0.061 | 0.62 | -0.035 | 0.77 | 0.125 | 0.30 | **-0.397** | **0.004** | -0.060 | 0.62 | -0.050 | 0.68 |
| *LEP*-CpG11 | 0.050 | 0.68 | 0.085 | 0.48 | -0.063 | 0.60 | 0.142 | 0.24 | -0.187 | 0.19 | 0.074 | 0.54 | 0.099 | 0.42 |
| *LEP*-CpG17 | -0.049 | 0.69 | 0.137 | 0.26 | 0.029 | 0.81 | 0.038 | 0.76 | -0.078 | 0.59 | -0.056 | 0.65 | 0.002 | 0.98 |
| *LEP*-Mean | 0.024 | 0.87 | 0.058 | 0.69 | -0.057 | 0.69 | -0.111 | 0.45 | **-0.285** | **0.05** | 0.105 | 0.47 | 0.025 | 0.86 |
| *ADIPOQ-CpGE3[a]* | 0.059 | 0.63 | 0.077 | 0.53 | -0.092 | 0.45 | 0.067 | 0.58 | 0.196 | 0.17 | 0.147 | 0.23 | 0.065 | 0.60 |
| **SAT** | | | | | | | | | | | | | | |
| *LEP*-CpG7 | -0.139 | 0.25 | 0.045 | 0.71 | 0.074 | 0.54 | 0.085 | 0.48 | 0.165 | 0.25 | 0.024 | 0.85 | 0.082 | 0.50 |
| *LEP*-CpG11 | -0.066 | 0.59 | 0.083 | 0.49 | -0.163 | 0.18 | 0.187 | 0.12 | 0.047 | 0.74 | 0.034 | 0.78 | 0.146 | 0.23 |
| *LEP*-CpG17 | -0.037 | 0.76 | 0.213 | 0.08 | -0.042 | 0.73 | -0.036 | 0.77 | 0.136 | 0.35 | -0.140 | 0.25 | 0.041 | 0.74 |
| *LEP*-Mean | -0.047 | 0.75 | 0.265 | 0.06 | -0.005 | 0.97 | 0.017 | 0.91 | 0.145 | 0.32 | 0.142 | 0.33 | 0.274 | 0.06 |
| *LEP mRNA levels* | -0.028 | 0.82 | -0.014 | 0.91 | 0.128 | 0.29 | -0.177 | 0.14 | -0.073 | 0.62 | 0.208 | 0.09 | 0.151 | 0.21 |
| *ADIPOQ*-CpGE1 | 0.045 | 0.71 | 0.163 | 0.18 | -0.212 | 0.08 | 0.090 | 0.46 | 0.186 | 0.20 | 0.008 | 0.95 | 0.042 | 0.73 |
| *ADIPOQ*-CpGE3 | 0.086 | 0.48 | **0.233** | **0.05** | -0.192 | 0.11 | 0.001 | 0.99 | 0.137 | 0.342 | -0.022 | 0.93 | -0.008 | 0.95 |
| *ADIPOQ*-Mean | 0.022 | 0.88 | **0.377** | **0.007** | -0.152 | 0.29 | 0.135 | 0.35 | 0.201 | 0.16 | -0.083 | 0.57 | -0.021 | 0.89 |
| *ADIPOQ mRNA levels* | 0.068 | 0.58 | -0.068 | 0.58 | -0.087 | 0.48 | 0.062 | 0.61 | -0.118 | 0.42 | 0.104 | 0.39 | 0.007 | 0.95 |
| **VAT** | | | | | | | | | | | | | | |
| *LEP*-CpG7 | 0.014 | 0.91 | 0.035 | 0.77 | **-0.237** | **0.05** | 0.182 | 0.13 | 0.043 | 0.77 | 0.008 | 0.95 | 0.127 | 0.29 |
| *LEP*-CpG11 | -0.100 | 0.41 | 0.067 | 0.58 | -0.141 | 0.24 | 0.145 | 0.23 | 0.106 | 0.47 | 0.097 | 0.42 | 0.199 | 0.10 |
| *LEP*-CpG17 | -0.156 | 0.20 | 0.153 | 0.21 | 0.128 | 0.29 | -0.029 | 0.81 | 0.142 | 0.33 | -0.037 | 0.76 | 0.061 | 0.62 |
| *LEP*-Mean | -0.161 | 0.26 | 0.196 | 0.17 | -0.019 | 0.897 | -0.048 | 0.74 | 0.133 | 0.36 | 0.093 | 0.52 | 0.178 | 0.22 |
| *LEP mRNA levels* | 0.019 | 0.88 | 0.117 | 0.33 | -0.112 | 0.36 | **0.311** | **0.009** | -0.114 | 0.43 | 0.178 | 0.14 | **0.310** | **0.009** |
| *ADIPOQ*-CpGE1 | 0.003 | 0.98 | 0.137 | 0.26 | -0.174 | 0.15 | -0.103 | 0.40 | 0.171 | 0.24 | -0.043 | 0.73 | -0.166 | 0.17 |
| *ADIPOQ*-CpGE3 | -0.083 | 0.49 | 0.110 | 0.37 | -0.024 | 0.85 | -0.121 | 0.32 | 0.121 | 0.40 | 0.012 | 0.93 | -0.107 | 0.38 |
| *ADIPOQ*-Mean | -0.072 | 0.62 | 0.176 | 0.22 | -0.004 | 0.98 | -0.064 | 0.66 | 0.161 | 0.26 | 0.013 | 0.93 | -0.146 | 0.31 |
| *ADIPOQ mRNA levels* | -0.083 | 0.50 | -0.104 | 0.40 | -0.084 | 0.50 | 0.098 | 0.43 | -0.213 | 0.5 | -0.003 | 0.98 | 0.017 | 0.89 |

TG, triglycerides*;* TC, total cholesterol; HDL-C, high-density lipoprotein-cholesterol; CRP, C-reactive protein; SBP, systolic blood pressure, DBP, diastolic blood pressure.
[a]Results obtained after rank transformation of the variable; [b]Results obtained after $log_{10}$-transformation
Values in bold type are statistically significant ($p \leq 0.05$)
