# Supplementary material for: Leptin and adiponectin DNA methylation levels in adipose tissues and blood cells are associated with BMI, waist girth and LDL-cholesterol levels in severely obese men and women
Source: BMC Med Genet. 2015 May 1;16:29. doi: 10.1186/s12881-015-0174-1 (PMC4631085; doi:10.1186/s12881-015-0174-1)
Supplement: Additional file 3: — CpG island within LEP gene proximal promoter region. The CpG sites are numbered according to Bouchard et al. 2010 [23]. The underlined CpG sites were epigenotyped in the current study. Associations between LEP DNA methylation levels, anthropometric variables and obesity-related complications were assessed at CpG sites for which DNA methylation values are in bold type. The sequence (−11131 to −10563) was numbered relative to the first leptin AUG codon (UCSC Genome Browser (Human Feb. 2009)). [file 12881_2015_174_MOESM3_ESM.pdf]

Additional File 3. PCR and pyrosequencing primers for *LEP* gene proximal promoter CpG island amplification and pyrosequencing.

Target CpGs	Target sequence (5' to 3')	Chromosomal Region[a]	Target sequence after bisulfite conversion (5'to 3')	PCR and pyrosequencing primers (5' to 3')	Length (bp)
CpG3 to CpG7	TC[7]GTAGGAATC[6]GCAGC[5]GC CARC[4]GGTTGCAAGGTAAGG CCCC[3]GGC[2]GC[1]G	chr7:127 881 330 to chr7:127 881 374	TC[7]GTAGGAATC[6]GTAGC[5]GT TARC[4]GGTTGTAAGGTAAGG TTTC[3]GGC[2]GC[1]G	F: GGGGAGGGTAGGTATGGAG<br>R: CCCTACATCCCTCCTAACT<br>seq: GGGTAGGTATGGAGT	162
CpG11 to CpG17	C[17]GGGGC[16]GGGAGCTGGC[15] GCTAGAAATGC[14]GCC[13]GGG GCCTGC[12]GGGGCAGTTGC[11]G CAAGTTGTGATC[10]GGGCC[9]G CTATAAGWGGGGC[8]G	chr7:127 881 230 to chr7:127 881 316	C[17]GGGGC[16]GGGAGTTGGC[15] GTTAGAAATGC[14]GTC[13]GGGG TTTGC[12]GGGGTAGTTGC[11]GT AAGTTGTGATC[10]GGGTC[9]GT TATAAGWGGGGC[8]G	F:ATTTTAGGGAGGTATTTAAGGGTG<br>R:ACCATTCCTACCAAACTCCATACCTACCC<br>seq: GGTAAGTAGTTATTTTGAGGG	239
CpG23 to CpG31	GC[31]GC[30]GC[29]GTGGCTCCTGG M[28]GC[27]GCC[26]GAGGCCCTCCC TC[25]GAGGCCCC[24]GC[23]GAG	chr7:127 881 125 to chr7:127 881 173	GC[31]GC[30]GC[29]GTGGTTTTTGG M[28]GC[27]GTC[26]GAGGTTTTTTT TC[25]GAGGTTTC[24]GC[23]GAG	F:GAGTTTTTGGAGGGATATTAAGGA<br>R:CAACTCCCCCCCCCCCCCTCAAAATAA<br>seq: GGGAGGTATTTAAGGG	194

The CpG sites are numbered according to Bouchard *et al.* 2010 [23]. The underlined CpG sites were epigenotyped in the current study
T in red font are cytosines that have been converted to thymine after Nabis treatment of DNA
F; Forward. R; Reverse. Seq; Sequencing
The primers were designed from bisulfite converted sequence using Pyromark Assay Design software (version 2.0.1.15; Qiagen).
[a]UCSC Genome Browser (Human Feb. 2009: NM_000230)
