# Supplementary material for: Leptin and adiponectin DNA methylation levels in adipose tissues and blood cells are associated with BMI, waist girth and LDL-cholesterol levels in severely obese men and women
Source: BMC Med Genet. 2015 May 1;16:29. doi: 10.1186/s12881-015-0174-1 (PMC4631085; doi:10.1186/s12881-015-0174-1)
Supplement: Additional file 4: — CpG islands analysed within ADIPOQ gene locus. CpG islands A and C were found to be hypomethylated and hypermethylated respectively and were not analysed. Associations between ADIPOQ DNA methylation levels, anthropometric variables and obesity-related complications were assessed at CpG sites for which DNA methylation values are in bold type. [file 12881_2015_174_MOESM4_ESM.pdf]

Additional File 4. PCR and pyrosequencing primers for *ADIPOQ* gene CpG islands amplification and pyrosequencing.

Target Region	Target sequence (5' to 3')	Chromosomal Region[a]	Target sequence after NaBis treatment (5'to 3')	PCR and pyrosequencing primers (5' to 3')	Length PCR (bp)
CpG Island A AdipoA3	TGCGGTCGCCCCGGCTCC CCGGCTCCAGAGCGCCCG CATTCCCGRGAGAGGCG ATGTGGGGGCCCGGG	Chr3:186 545 134 Chr3:186 545 202	TGCGGTCGTTTCGGTTTT TCGGTTTTAGAGCGTTCG TATTTTCGAGAGAGGCGA TGTGGGGGTTCGGG	F: GGATTTTTATTTAGGAGAGTTGTTTT R: ACCCTAAACCTCCCCTTTCTACC seq: ATTTTTATTTAGGAGAGTTGTTTTT	161
CpG Island C AdipoC1	CGCGGTGGCTCACGCCTG TCATTCCAGCACTTTGGG AGGCCG	Chr3:186 557 086 Chr3:186 557 128	CGCGGTGGTTTACGTTTG TTATTTTAGTATTTTGGG AGGTCG	F: GGTGGTAGGAGGTGATAGTTTAA R: ACTCCCCACCTCAAATAATCCAC seq: GAAATGTTTTTTTGGTTAGG	199
CpG Island E AdipoE2	GCGTACGTATGTGGATGT GTGGATGTGGTGTGTGGK TGTGCGCGT	Chr3:186 562 910 Chr3:186 562 954	GCGTACGTATGTGGATGT GTGGATGTGGTGTGTGGG TGTGCGCGT	F: TGGTGAGTGGGATGTTTTGTTTTA R: ACACACACCTCCACCTAT seq: CACACACCTCCACCTATA	180

The CpG island are numbered according to Bouchard *et al*. 2012 [24]. The underlined CpG sites were epigenotyped in the current study.
T in red font are cytosines that have been converted to thymine after Nabis treatment of DNA
F; Forward. R; Reverse. Seq; Sequencing
The primers were designed from bisulfite converted sequence using Pyromark Assay Design software (version 2.0.1.15; Qiagen).
[a]UCSC Genome Brower (Human Feb, 2009: NM_001177800)
