# Supplementary material for: Leptin and adiponectin DNA methylation levels in adipose tissues and blood cells are associated with BMI, waist girth and LDL-cholesterol levels in severely obese men and women
Source: BMC Med Genet. 2015 May 1;16:29. doi: 10.1186/s12881-015-0174-1 (PMC4631085; doi:10.1186/s12881-015-0174-1)
Supplement: Additional file 5: — Pearson correlation coefficients between LEP and ADIPOQ DNA methylation and mRNA levels in blood, subcutaneous (SAT) and visceral adipose tissue (VAT) and anthropometric variables (adjusted for age and sex) (n = 73). [file 12881_2015_174_MOESM5_ESM.pdf]

-11131

...ATGCCCCAGCCCGCTCCGCGGAGCCCCYCACAGCCACCCCCGCCCCGACCGCGCCCCGCGCGGCTCGAAGCACCTTCCCAAGGGGCTGGTCCTTGCGCCATAGTCG

31 30 29 28 27 26

CGCCGGAGCCTCTGGAGGGACATCAAGGATTTCTCGCTCCTACCAGCCACCCCCAAATTTTYGGGAGGTACCCAAGGGTGCGCGCGTGGCTCCTGGMGCGCCGAGGCC

25 24 23 22 21 20 19 18 17 16 15 14 13

CTCCCTCGAGGCCCCGCGAGGTGCACACTGCSGGCCCAGGGCTAGCAGCCGCCCGGCACGTCGCTACCCTGAGGGGCGGGGCGGGAGCTGGCGCTAGAAATGCGCC

12 11 10 9 8 7 6 5 4

GGGGCCTGCGGGGCAGTTGCGCAAGTTGTGATCGGGCCGCTATAAGWGGGGCGGGCAGGCATGGAGCCCCGTAGGAATCGCAGCGCCARCGGTTGCAAGGTAAGGC

rs2167270

3 2 1

CCCGGCGCGCTCCTTCCTCCTTCTCTGCTGGTCTTTCTTGGCAGGCCACAGGGCCCCACACAACTCTGGATCCCGGGGAAACTGAGTCAGGAGGGATGCAGGGCGGAT

GGCTTAGTTCTGGACTATGATAGCTTTGTACCGAG...

-10563

	Mean DNA methylation levels ± SD (%)		
	SAT	VAT	Blood
CpG3	34.6 ± 5.2	35.8±4.7	34.6±6.9
CpG4	18.4 ± 3.5	18.8±3.2	29.2±7.8
CpG5	17.5 ± 3.0	17.0±2.8	24.4±6.5
CpG6	16.5 ± 3.6	16.5±3.2	23.2±6.5
CpG7	**16.4 ± 3.4**	**15.3±2.5**	**21.0±6.0**
CpG11	**24.5 ± 2.8**	**23.9±4.1**	**40.5±8.5**
CpG12	8.6 ± 1.4	7.6±1.2	10.0±2.7
CpG13	9.7 ± 1.7	6.8±1.2	7.9±2.5
CpG14	8.6 ± 1.4	6.1±1.1	8.8±2.7
CpG15	7.7 ± 1.3	6.3±0.9	9.0±2.3
CpG16	6.4 ± 1.3	5.7±1.2	10.3±3.7
CpG17	**10.7 ± 2.1**	**10.0±2.1**	**17.7±5.2**
CpG23	8.8 ± 1.8	9.5±1.9	16.7±4.7
CpG24	16.8 ± 2.7	15.9±3.1	28.6±7.6
CpG25	17.7 ± 2.9	14.5±2.5	21.2±4.4
CpG26	15.8 ± 3.0	14.8±3.1	23.1±6.3
CpG27	11.0 ± 1.9	11.7±2.5	19.8±4.4
CpG28	9.7 ± 2.9	10.5±3.4	20.0±7.3
CpG29	15.9 ± 2.5	17.3±3.2	28.4±7.9
CpG30	17.4 ± 3.2	17.6±3.9	31.4±8.3
CpG31	21.1 ± 3.6	21.7±4.0	35.2±7.9
Mean	**14.9 ± 2.0**	**14.4±1.9**	**22.0±4.4**
