# Supplementary material for: Leptin and adiponectin DNA methylation levels in adipose tissues and blood cells are associated with BMI, waist girth and LDL-cholesterol levels in severely obese men and women
Source: BMC Med Genet. 2015 May 1;16:29. doi: 10.1186/s12881-015-0174-1 (PMC4631085; doi:10.1186/s12881-015-0174-1)
Supplement: Additional file 6: — Pearson correlation coefficients between LEP and ADIPOQ DNA methylation and mRNA levels in blood, subcutaneous (SAT) and visceral adipose tissues (VAT) and cardiometabolic risk factors (adjusted for age, sex and waist circumference) (n = 73). [file 12881_2015_174_MOESM6_ESM.pdf]

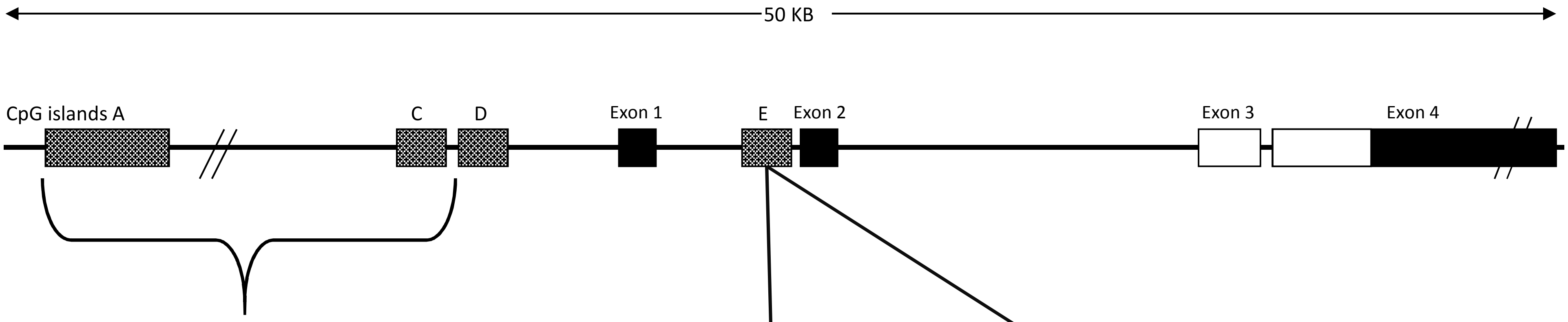

	Mean DNA methylation levels ± SD (%)		
	**SAT**	**VAT**	**Blood**
CpG island A	3.0 ± 1.0	2.5 ± 0.8	2.5 ± 1.1
CpG island C	95.9 ± 3.9	95.9 ± 3.5	94.0 ± 6.7

	Mean DNA methylation levels ± SD (%)		
	**SAT**	**VAT**	**Blood**
CpGE1	**84.2 ± 3.3**	**86.7 ± 3.5**	95.1 ± 2.5
CpGE2	91.1 ± 2.2	91.0 ± 3.1	96.3 ± 1.7
CpGE3	**68.1 ± 3.7**	**70.6 ± 4.2**	**82.0 ± 3.7**
Mean	**81.2 ± 2.7**	**82.8 ± 3.3**	91.2 ± 2.4
